# Mindfulness and progressive muscle relaxation as standardized session‐introduction in individual therapy: A randomized controlled trial

**DOI:** 10.1002/jclp.22695

**Published:** 2018-10-08

**Authors:** Johannes Mander, Paul Blanck, Andreas B. Neubauer, Paula Kröger, Christoph Flückiger, Wolfgang Lutz, Sven Barnow, Hinrich Bents, Thomas Heidenreich

**Affiliations:** ^1^ Center for Psychological Psychotherapy University of Heidelberg Heidelberg Germany; ^2^ German Institute for International Educational Research (DIPF) Frankfurt am Main Germany; ^3^ Department of Clinical Psychology and Psychotherapy University of Zürich Zürich Switzerland; ^4^ Department of Clinical Psychology and Psychotherapy University of Trier Trier Germany; ^5^ Department of Clinical Psychology and Psychotherapy University of Heidelberg Heidelberg Germany; ^6^ Department of Social Work, Health and Nursing University of Applied Sciences Esslingen Esslingen am Neckar Germany

**Keywords:** component study, mindfulness, psychotherapy process, randomized controlled trial (RCT), therapeutic alliance

## Abstract

**Objective:**

There is scarce research on the effects of mindfulness in individual therapy. As many practitioners integrate mindfulness exercises into individual therapy, empirical evidence is of high clinical relevance.

**Method:**

We investigated the effects of a session‐introducing intervention with mindfulness elements (SIIME) in a randomized, controlled design. The effects of SIIME on therapeutic alliance and symptomatic outcome were compared with progressive muscle relaxation (PMR) and treatment‐as‐usual (TAU) control conditions. The sample comprised 162 patients with anxiety and depression.

**Results:**

Multilevel modeling revealed a significant symptom reduction and significant increase of alliance over the course of therapy. There were no significant time–condition interactions on outcome and alliance, indicating the comparable efficiency of all three treatment conditions.

**Conclusions:**

We found no advantage of SIIME versus PMR and TAU. Add‐on mindfulness might not improve individual therapy related to alliance and outcome.

## 1 INTRODUCTION

Anxiety and depression are the two most frequent mental health problems (Whiteford et al., 2013). With a lifetime prevalence of 20% for anxiety and 30% for depression, these mental disorders have high economic costs (Somers, Goldner, Waraich, & Hsu, 2006; Waraich, Goldner, Somers, & Hsu, 2004). Mindfulness, a specific form of attention that is nonjudgmental, purposeful, and focused on the present moment (Bishop et al., 2004; Kabat‐Zinn, 2013), is well established in modern cognitive behavioral therapy (CBT) as a treatment element for anxiety and depression (Khoury et al., 2013). Mindfulness‐based interventions (MBIs) have been intensively investigated in manualized group therapy programs with mindfulness‐based stress reduction (MBSR; Kabat‐Zinn, 2013) and mindfulness‐based cognitive therapy (MBCT; Segal, Williams, & Teasdale, 2013) as its most prominent applications (Michalak, Schultze, Heidenreich, & Schramm, 2015). Both MBIs are offered in eight weekly 2‐hr classes, where patients learn meditations that include a sustained attentional focus on body, breath, and thoughts (Huijbers et al., 2016). Recent meta‐analyses show medium‐to‐large effect sizes on symptoms of anxiety and depression for manualized MBIs (anxiety: *g* = 0.64–1.00; depression: *g* = 0.53–0.95), demonstrating their clinical efficacy (Goldberg et al., 2018; Hofmann, Sawyer, Witt, & Oh, 2010; Khoury et al., 2013). In these meta‐analyses, mindfulness‐related variables (dispositional mindfulness, intensity of mindfulness practice, mindfulness experience, allegiance) and demographic and diagnostic variables (sex, age, gender, diagnosis, comorbidities) have been investigated as potentially relevant moderating variables. Only mindfulness‐related variables proved to be significant moderators of outcome effects of MBIs. MBIs consist of several components, including different mindfulness and yoga interventions, psychoeducation, and group‐related factors, such as group cohesion and social support (Shallcross et al., 2015). First, dismantling studies of MBIs in group settings found no advantage related to clinical outcomes of the whole MBCT package compared with a control group, where the mindfulness exercise component was removed (Shallcross et al., 2015; Williams et al., 2014). On the other hand, mediation studies found positive outcome effects of the mindfulness component (Kuyken et al., 2010; van der Velden et al., 2015). Consequently, it remains unclear whether mindfulness exercises are the specific core component for therapeutic change (Van Dam et al., 2018).

Many clinicians in routine care apply mindfulness exercises in individual therapy (Cigolla & Brown, 2011; Demarzo et al., 2015; Dimidjian & Segal, 2015) and already do so during their training (Ivanovic, Swift, Callahan, & Dunn, 2015; Mander et al., 2017a). Typically, therapists apply only single mindfulness exercises (often short exercises like brief sitting meditation or breathing space) and not the whole MBCT program in individual therapy sessions (Cigolla & Brown, 2011; Horst, Newsom, & Stith, 2013; Michalak, Steinhaus, & Heidenreich, 2018). Empirical evidence on the effects of mindfulness exercises in individual therapy settings is scarce (Kuyken et al., 2016). Based on the recommendations of the National Institute of Health stage model (Onken, Carroll, Shoham, Cuthbert, & Riddle, 2014), studying mindfulness exercises in individual therapy in routine care has been identified as an important research gap (Dimidjian & Segal, 2015). First studies demonstrate the positive effects of mindfulness on therapeutic alliance in cross‐sectional designs (Dunn, Callahan, Swift, & Ivanovic, 2013; Horst et al., 2013), but lack evidence on symptomatic outcome in longitudinal designs (Goyal et al., 2014). A pilot study comparing MBCT in group and individual therapy showed significant symptom improvement in individual MBCT that was equally strong as in group MBCT (Schroevers, Tovote, Snippe, & Fleer, 2016). However, this study investigated the effects of the whole MBCT package and did not directly target the mindfulness exercise component (Britton et al., 2017). Furthermore, a meta‐analysis showed that mindfulness exercises as a stand‐alone intervention have beneficial effects on anxiety and depression in healthy subjects (Blanck et al., 2017). The next step of research would be to investigate the integration of mindfulness exercises as an add‐on component in individual therapy (Cuijpers, Cristea, Karyotaki, Reijnders, & Hollon, 2017).

A core intervention of MBCT is the breathing space (Segal et al., 2013). The breathing space is a mini‐meditation that can help advance into the present moment. In MBCT, the breathing space is used to structure each day and to deal with difficult situations by coming into a state of present moment awareness and then applying relevant coping strategies (Michalak, Heidenreich, & Williams, 2012). Thus, the breathing space could also be applied in individual therapy as a session‐introducing intervention to come into a state of present moment awareness at the beginning of therapy sessions (Mander et al., 2015). According to the model of Kristeller and Johnson (2005), mindfulness awareness leads to stronger self‐compassion and increased empathy for others, thereby strengthening the therapeutic alliance. Consequently, it should be possible to more effectively apply therapeutic interventions in the current session (Bruce, Manber, Shapiro, & Constantino, 2010). Based on this model and in line with preliminary research evidence, session‐introducing mindfulness should ultimately strengthen therapeutic alliance and increase the importance of the alliance for symptomatic outcome (Dunn et al., 2013; Horst et al., 2013). However, it is crucial to compare this approach with active control groups that include exercises without the hypothesized effective ingredients of MBIs (Britton et al., 2017; Shallcross et al., 2015). A strong and empirically validated exercise that does not include the hypothesized effective components of mindfulness is Jacobson’s (1938) progressive muscle relaxation (PMR; Semple, 2010). More specifically, similar to MBIs, PMR is also applied as a stress‐management technique (Goyal et al., 2014). Whereas the focus of MBIs is to observe moment‐to‐moment experiences as they are (Williams et al., 2014), the objective of PMR is to increase sensations of physical relaxation (Gao, Curtiss, Liu, & Hofmann, 2017). Of additional importance, MBIs and PMR have been applied in multiweek clinical interventions, but they can also be used as stand‐alone interventions that can be incorporated into single therapy sessions (Schroevers et al., 2016; Semple, 2010).

Recently, it has been highlighted that randomized controlled trials (RCTs) should be conducted in routine individual therapy to address issues of external validity (Flückiger et al., 2016; Lutz et al., 2015; McHugh & Barlow, 2010). As outlined above, it is of clinical relevance to investigate mindfulness exercises in individual therapy (Dimidjian & Segal, 2015; Michalak et al., 2015). Consequently, we designed an RCT under routine conditions for patients with anxiety and depression and investigated whether a session‐introducing version of the breathing space adapted for individual therapy can improve treatment. We matched overall therapy time by directly integrating mindfulness into individual therapy to not confound potential effects with extra treatment time (Britton et al., 2017). Specifically, we examined the effects of a 5‐min session‐introducing intervention with mindfulness elements (SIIME) practiced together by outpatients and therapists at the beginning of the 25 therapy sessions on (a) clinical outcomes assessed four times during treatment, and (b) therapeutic alliance measured on a session‐to‐session basis. Before treatment, patients were randomized into a treatment‐as‐usual+mindfulness intervention group (TAU+M) practicing SIIME, a TAU+PMR‐control group practicing a session‐introducing short form of PMR, or a TAU control group without standardized session‐introducing intervention. Before the start of the intervention study, all therapists participated in two 6‐week workshop programs, one for mindfulness and one for PMR. In line with the findings outlined above, we investigated the following hypotheses: There will be stronger reductions in clinical symptomatology of patients in TAU+M compared with the TAU+PMR and TAU conditions (Hypothesis 1). Both patients and therapists of the TAU+M will experience stronger increases of the therapeutic alliance compared with the TAU+PMR and TAU conditions (Hypothesis 2). We hypothesized that the mindfulness condition will be a significant moderator of the alliance–outcome association (Hypothesis 3). We further investigated whether relevant moderators that have been investigated in the mindfulness group literature generalize to individual therapy settings. We hypothesized in line with the above‐mentioned meta‐analyses on group MBIs that mindfulness‐related variables will increase mindfulness effects (Hypothesis 4a) and that demographic and diagnostic variables will not be significant moderators (Hypothesis 4b).

## 2 METHOD

### 2.1 Participants

The study was conducted between November 2014 and January 2018 at a university outpatient training center for CBT. The training followed the standard procedures of CBT formation in Germany. Specifically, the 48 study therapists performed 18 months of internships in psychiatric and psychosomatic hospitals before starting with their outpatient individual therapies for the study. Furthermore, they received a mean of 362.15 hr (*SD* = 123.42, target = 600 hr) of theory, 120 hr of self‐experience, and performed on average 88.79 hr (*SD* = 103.75, target = 600 hr with 150 hr of supervision) of outpatient individual therapy before the start of the study. Participating trainee therapists had predominantly little mindfulness experience and did not have a formal mindfulness qualification (e.g., MBCT/MBSR teacher). Inclusion criteria for patients were (a) a primary anxiety or depressive disorder diagnosis; (b) age between 18 and 65 years; (c) fluency and literacy in German; and (d) informed consent. Exclusion criteria were (a) psychotic disorders; (b) a major medical illness (e.g., cancer); and (c) current suicidal risk. Comorbidities with disorders not on the exclusion list were generally not considered limitations to entering the study, as long as anxiety and depressive disorders were of primary concern. We chose these two mental disorders because of patients being treated at German university therapy‐training centers; approximately 30% suffer from a primary anxiety disorder, and approximately 40% suffer from a primary major depression (Nelson & Hiller, 2013). Hence, our results are of relevance for a majority of patients treated in outpatient training centers. Altogether, 162 patients treated by 48 therapists participated in the study. For sample characteristics see Table 1.

**Table 1:** Patient and therapist sample: Baseline characteristics

Characteristic	Entire sample (*n = *162)	TAU+M (*n = *54)	TAU+PMR (*n = *54)	TAU (*n = *54)	*p* value
Age, *M* (*SD*)	35.07 (12.69)	37.20 (12.47)	32.59 (12.35)	35.43 (13.05)	0.16^a^
Female sex, *n* (%)	98 (60.5)	36 (66.7)	33 (61.1)	29 (53.7)	0.39^b^
Qualifications for university entrance, *n* (%)	72 (44.4)	22 (40.7)	27 (50.0)	23 (42.6)	0.59^b^
Formal job qualification, *n* (%)	100 (61.7)	39 (72.2)	31 (57.4)	30 (55.6)	0.15^b^
Employed, *n* (%)	119 (73.5)	43 (80.0)	42 (77.8)	34 (63.0)	0.10^b^
In a relationship, *n* (%)	95 (58.6)	33 (61.1)	34 (63.0)	28 (51.9)	0.45^b^
Primary diagnosis: depression, *n* (%)	93 (57.4)	32 (59.2)	31 (57.4)	30 (55.6)	0.93^b^
Primary diagnosis: anxiety, *n* (%)	69 (42.6)	22 (40.7)	23 (42.6)	24 (44.4)	0.93^b^
At least one comorbid disorder, *n* (%)	71 (43.8)	28 (51.9)	23 (42.6)	20 (37.0)	0.29^b^
Prior psychotherapy experience, *n* (%)	91 (56.1)	32 (59.3)	28 (51.9)	31 (57.4)	0.72^b^
Receiving psychotropic medication, *n* (%)	77 (47.5)	23 (42.6)	24 (44.4)	30 (55.6)	0.35^b^
Prior mindfulness experience, *n* (%); years (*SD*)	56 (34.6); 0.68 (1.81)	21 (38.9); 0.73 (1.78)	18 (33.3); 0.84 (2.23)	17 (31.5); 0.47 (1.30)	0.70^b^; 0.56^a^
Prior progressive muscle relaxation experience, *n* (%); years (*SD*)	56 (34.6); 0.90 (2.30)	20 (37.0); 0.92 (2.53)	20 (37.0); 1.18 (2.69)	16 (29.6); 0.61 (1.52)	0.65^b^; 0.43^a^
Therapists (*n = *48)					
Female sex, *n* (*%*)		42 (87.5)			
Age, *M* (*SD*)		30.42 (5.22)			
Treated outpatients, *M* (*SD*)		6.44 (5.23)			
Conducted therapy sessions, *M* (*SD*)		88.79 (103.75)			
Prior mindfulness experience *n* (*%*); years (*SD*); weekly practice (*%*)		36 (75.0); 2.41 (3.52); 12 (25.0)			
Prior progressive muscle relaxation experience *n* (*%*); years (*SD*); weekly practice (*%*)		41 (85.4); 2.54 (2.24); 2 (4.2)			

*Note*. M: mindfulness; PMR: progressive muscle relaxation; TAU: treatment‐as‐usual.

^a^ By analysis of variance.

^b^ By the *χ* ^2^ test.

### 2.2 Measures

#### 2.2.1 Diagnoses

The Structured Clinical Interview (SCID), according to the DSM‐5 criteria (American Psychiatric Association, 2013), was applied to establish the diagnoses. Experienced clinicians trained in SCID workshops acted as diagnosticians. SCID assessments were conducted at baseline and at the end of treatment. All diagnostic interviews were video recorded. Twenty percent (*n* = 33) were randomly selected and rated by a second diagnostician for reliability information. The agreement between diagnosticians was high (к = 0.93, *p* < 0.001).

#### 2.2.2 Primary outcome measures

The Brief Symptom Inventory (BSI; Derogatis & Melisaratos, 1983; Franke, 2000) is a commonly administered self‐report measure that detects general clinical symptoms and consists of 53 items, forming 9 subscales: somatization, obsessive‐compulsive, interpersonal sensitivity, depression, anxiety, hostility, phobic anxiety, paranoid ideation, and psychoticism, which are rated on a five‐step scale. The nine subscales can be integrated into a Global Severity Index (GSI) with an excellent internal consistency of *α* = 0.90 (current sample: 0.95 ≤ *α* ≤ 0.97) and a good predictive validity of 0.30 ≤ *r* ≤ 0.72 (Derogatis & Fitzpatrick, 2004; Derogatis & Melisaratos, 1983). According to the authors, the GSI is the single best indicator of current distress levels and should be applied when a summary measure of clinical symptoms is required (Derogatis & Fitzpatrick, 2004). The GSI was applied as the primary outcome in the current study.

#### 2.2.3 Secondary outcome measures

The Working Alliance Inventory‐Short Revised (WAI‐SR; Hatcher & Gillaspy, 2006) was applied to assess the quality of the therapeutic alliance. The WAI‐SR is a 12‐item self‐report instrument with patient and therapist perspectives rated on a seven‐point scale (range: 1–7). It includes three subscales (bond, goals, and tasks) that can be integrated into one global alliance score (Horvath, Del Re, Flückiger, & Symonds, 2011). The WAI‐SR is considered the gold standard for alliance assessment, has excellent psychometric properties with 0.81 ≤ *α* ≤ 0.92 (current sample: 0.87 ≤ *α* ≤ 0.96), and is outcome predictive with *r* = 0.28, as has been demonstrated in meta‐analyses (Horvath et al., 2011).

The Beck Anxiety Inventory (BAI; Beck, Epstein, Brown, & Steer, 1988; Margraf & Ehlers, 2007) was applied to assess general anxiety symptoms of the patients. It consists of 21 items that revealed an internal consistency of *α* = 0.90 (current sample: 0.90 ≤ *α* ≤ 0.92), a split‐half reliability of *r* = 0.70, a retest reliability of *r* = 0.75, and convergent validities of 0.50 ≤ *r* ≤ 0.61 (Beck et al., 1988; Margraf & Ehlers, 2007).

The Beck Depression Inventory‐II (BDI‐II; Beck, Steer, & Brown, 1996; Hautzinger, Keller, & Kühner, 2006) was applied to assess severity of depressive symptoms. It consists of 21 items and revealed an internal consistency of *α* = 0.88 (current sample: 0.91 ≤ *α* ≤ 0.95), a split‐half reliability of *r* = 0.72, a retest reliability of *r* = 0.75, and convergent validities of 0.71 ≤ *r* ≤ 0.89 (Beck et al., 1996; Hautzinger et al., 2006).

The Kentucky Inventory of Mindfulness Skills (KIMS; Baer, Smith, & Allen, 2004) was applied to assess mindfulness experiences in everyday life. It consists of 39 items (range: 0–4) that are rated on a five‐step scale with four subscales: observing, describing, acting with awareness, and accepting without judgment. It has factor loadings of 0.41 ≤ *λ* ≤ 0.86; internal consistencies of 0.83 ≤ *α* ≤ 0.91 (current sample: 0.81 ≤ *α* ≤ 0.90), and is outcome predictive (Baer et al., 2004; Ströhle, Nachtigall, Michalak, & Heidenreich, 2010).

The Hamilton Rating Scale for Depression (HAM‐D; Hamilton, 1967) was used as an interview‐based measure to assess the severity of depressive symptoms with 17 items. Internal consistencies ranged from 0.73 ≤ *α* ≤ 0.81 (current sample: 0.71 ≤ *α* ≤ 0.80), intraclass correlations (ICCs) were 0.78 ≤ ICC ≤ 0.82, and cutoff to remission ≤ 6 (Moras, di Nardo, & Barlow, 1992). Patients were instructed not to mention their treatment condition at the beginning of each interview to maintain rater blindness.

The Hamilton Rating Scale for Anxiety (HAM‐A; Hamilton, 1959) was used as an interview‐based measure to assess the severity of anxiety symptoms with 14 items. Internal consistencies ranged from 0.77 ≤ *α* ≤ 0.81 (current sample: 0.79 ≤ *α* ≤ 0.82), ICCs were 0.74 ≤ ICC ≤ 0.96, and cutoff to remission ≤ 7 (Moras et al., 1992).

#### 2.2.4 Demographics, adherence, competence, and allegiance

Standard measures of the demographic data of patients were assessed at baseline. In addition, the intensity of the patients’ and therapists’ mindfulness and PMR experiences before the study, as well as the current intensity of patients’ and therapists’ mindfulness and PMR exercises conducted outside of the therapy sessions, was assessed. Furthermore, we assessed adherence to the interventions with self‐report and video‐based observer ratings. Patients’ and therapists’ self‐reported adherence was assessed with eight items (range: 0–4). The items assessed whether participants could adequately perform the different components of SIIME or PMR (e.g., *Were you able to focus on your breath during intervention?* W*ere you able to tense and relax both arms?*). In addition, observer adherence was assessed using video‐based analyses. The six items (range: 0–4) assessed whether participants conducted the interventions adequately (e.g., *participant took adequate body position*/*participant tightened and relaxed both arms*).

The Therapist Presence Inventory (TPI; Geller, Greenberg, & Watson, 2010) was applied to assess in‐session therapeutic presence operationalized as being fully in the present moment with an attitude of acceptance and openness (six items, range: 0–4). The TPI showed an internal consistency of *α* = 0.75 and demonstrated convergent validity (Geller et al., 2010).

The Practice Quality‐Mindfulness (PQ‐M; del Re, Flückiger, Goldberg, & Hoyt, 2013) was applied to assess the perceived quality of mindfulness implementation operationalized as perseverance in (a) receptive attention and (b) present‐moment attention (six items, range: 0–4). The measure showed internal consistencies of 0.72 ≤ *α* ≤ 0.87 and was outcome predictive (del Re et al., 2013).

The Cognitive Therapy Scale (CTS; Weck, Hautzinger, Heidenreich, & Stangier, 2010; Young & Beck, 1980) was applied to assess therapists’ competence based on observer ratings (14 items, range: 0–6). The interrater reliabilities were 0.73 ≤ ICC ≤ 0.95 (Weck et al., 2010). The interrater reliability of the CTS mean score between the two raters in the current sample was ICC (2,2) = 0.83; *p* < 0.001.

To test potential allegiance effects related to the session‐introducing interventions, we applied an adapted version of the allegiance scale developed by Falkenström, Markowitz, Jonker, Philips, and Holmqvist (2013). The scale consists of 10 items (range: 0–4) that assess the personal and professional attitude of therapists and patients towards the SIIME, PMR, and TAU session introduction (e.g., *I find it satisfying to work with SIIME*). Internal consistency was excellent with *α* = 0.97.

To test potential allegiance effects related to the offered treatment, we applied the credibility/expectancy questionnaire (Devilly & Borkovec, 2000). The six items (range: 1–9) of the measure assess treatment expectancy and rationale credibility. It has internal consistencies of 0.85 ≤ *α* ≤ 0.86 and a predictive validity of 0.26 ≤ *r* ≤ 0.68 (Devilly & Borkovec, 2000).

To assess the application of specific therapeutic techniques, we applied an instrument that measures therapists’ self‐report of the intensity of applied CBT techniques (Löffler et al., 2015). The subscales of the measure assess psychoeducation, behavioral activation, cognitive restructuring, exposure therapy, functional analysis, and relationship techniques (30 items, range: 0–4). It has factor loadings of 0.51 ≤ *λ* ≤ 0.88, internal consistencies of 0.60 ≤ *α* ≤ 0.77, and is outcome predictive (Löffler et al., 2015).

### 2.3 Procedures

**Figure 1:**
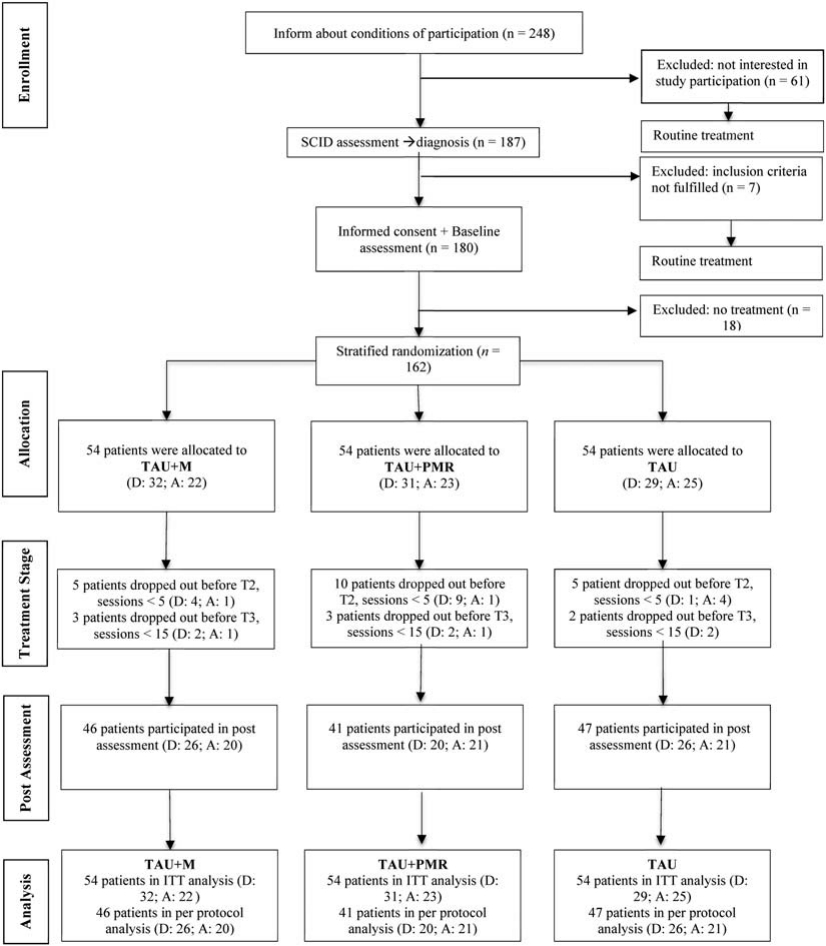
CONSORT flow diagram. A: anxiety; D: depression; ITT: intention‐to‐treat; M: mindfulness; PMR: progressive muscle relaxation; TAU: treatment‐as‐usual

This trial was registered at ClinicalTrials.gov (NTC02270073). Details on the study protocol are published elsewhere (Mander et al., 2015). The treatment duration was 25 weekly sessions (50 min per session). SCID, HAM‐A, and HAM‐D were conducted at baseline and after Session 25. Patient self‐ratings of clinical symptoms, mindfulness, and treatment integrity were assessed at baseline (Pre), after Session 5 (S5), after Session 15 (S15), and after Session 25 (S25). Therapeutic alliance was assessed after each session. All therapy sessions were videotaped and every fourth session was supervised by an accredited expert. Voluntary participation and written, informed consent were necessary conditions for participation in the study. The local ethics committee approved the study protocol in accordance with the Helsinki Declaration. Potential study participants were screened with a clinical telephone interview using a screening checklist based on the SCID. In case this prescreening indicated a main diagnosis of anxiety or depression, a face‐to‐face SCID was conducted. Eligible participants were assigned to one of the three treatment groups by a stratified randomization process. Patients were stratified into one of two categories: one group with the main diagnosis of an anxiety disorder and the other group with the main diagnosis of major depression. According to the CONSORT statement (Altman et al., 2001), randomization was conducted by an independent research assistant, whereas the other researchers had no access to the randomization list and process. The focus of the current study was on external validity and generalizability of the results to routine clinical practice. Consequently, according to the extension of the CONSORT statement concerning the criteria of a pragmatic, randomized, controlled trial (Zwarenstein et al., 2008), no blinding concerning treatment conditions was implemented in the current study. However, participants were blind concerning the specific study hypotheses. A flow diagram is depicted in Figure 1.

### 2.4 Interventions

#### 2.4.1 Workshop training

As study preparation, all participating trainee therapists received two workshop programs (one for mindfulness, one for PMR). Both workshop programs were offered by an expert with over 20 years of mindfulness and PMR experience. Both programs were based on the following procedure: a first workshop included background information and practice of relevant exercises. Then, a 6‐week home practice interval followed. In the second workshop, home practice experiences were discussed. Ideas on how to integrate the exercises into therapy routine and to continue the home practice were the focus. The workshops were well accepted and participating therapists reported positive experiences in qualitative interviews (for further details, see Mander et al., 2017a).

#### 2.4.2 Routine therapy

Treatment consisted of CBT training therapy based on evidence‐based practice protocols (Hofmann, Asnaani, Vonk, Sawyer, & Fang, 2012). Basic interventions included psychoeducation, as well as cognitive (e.g., functional analysis, cognitive restructuring) and behavioral (e.g., behavioral activation and exposure therapy) techniques. As we investigated training therapy under routine conditions, therapy was not based primarily on specific CBT treatment manuals, but rather on individual case conceptions as developed by trainees in collaboration with their supervisors. This procedure served to guarantee the optimal adaptation of therapeutic interventions on the individual needs of the patients. Patients received the same treatment in all three study conditions. The only difference was the intervention in the first 5 min (TAU+M vs. TAU+PMR vs. TAU).

#### 2.4.3 Treatment‐as‐usual+mindfulness

In the TAU+M experimental condition, each of the 25 sessions started with the audiotaped SIIME (duration = 5 min, wording listed in the Supporting Information Appendix), spoken by an experienced mindfulness expert. The SIIME is an adapted version of the breathing‐space exercise (Michalak et al., 2012; Segal et al., 2013) from the MBCT program. The breathing space follows an hourglass structure of attention with the three facets: wide angle (becoming aware of current thoughts, feelings, and body sensations), narrow angle (focus attention on breathing), and wide angle (expanding attention to the body as a whole). An iterative process in multiple steps with feedback from 20 patient‐therapist dyads and 5 mindfulness experts resulted in the following adaptions for the routine care setting: the SIIME rather follows the typical attention structure of sitting meditations in the MBCT program and consists only of the two attention components, narrow angle (focusing attention on breathing) and wide angle (expanding attention to the body as a whole), with an attitude of acceptance and openness in the here and now. According to the feedback, this structure was easier for participants with little mindfulness experience.

#### 2.4.4 Treatment‐as‐usual+progressive muscle relaxation

In the TAU+PMR control condition, each of the 25 sessions started with the audiotaped session‐introducing PMR intervention (duration = 5 min, wording listed in the Supporting Information Appendix), spoken by an experienced expert. We chose PMR as the active control group for the following reasons: on the one hand, PMR is a broadly accepted and an easy‐to‐implement relaxation exercise. On the other hand, it does not include the hypothesized specific effective ingredients of the SIIME (observing moment‐to‐moment experiences with an attitude of acceptance and openness). The PMR control condition was developed based on the same iterative procedure as the SIIME.

#### 2.4.5 Treatment‐as‐usual

In the TAU condition, no standardized session introduction was applied. Participants were free to arrange the start of therapy the way that seemed most consistent for them. Typically, CBT routine introductions (e.g., agenda setting) were applied.

#### 2.4.6 Rationale

Before conducting the first intervention, a standardized rationale that provided clinical reasons for the relevant condition (TAU+M, TAU+PMR, or TAU) was presented by the therapists (wording listed in the Supporting Information Appendix). In addition, all components of the relevant intervention were explained and practiced. Furthermore, in the first therapy sessions, a brief inquiry (brief exploration of experiences during the exercises) was conducted after SIIME and PMR. If required, the inquiry was conducted in later therapy sessions too.

### 2.5 Statistical analyses

Power analyses with G*Power (Faul, Erdfelder, Lang, & Buchner, 2007) for the detection of small effects (Cohen’s *f* = 0.12) for the interaction between time (Pre, S5, S15, and S25) and treatment condition (TAU+M vs. TAU+PMR vs. TAU; analysis of variance [ANOVA] repeated measures, within‐between‐interaction, *α* = 0.05, power (1 − β) = 0.80, number of groups = 3, number of measurements = 4, pre–post correlation *r* = 0.5, nonsphericity correction = 1) for the GSI primary outcome resulted in a sample size of 123 patients. Although this should only be seen as a proxy for the power of the reported multilevel models (MLMs), previous research showed that power in MLM is comparable to (or even higher than) power in an ANOVA design (Baldwin, Imel, Braithwaite, & Atkins, 2014; Quené & van den Bergh, 2004). The planned sample size should, therefore, be considered a conservative estimate required to detect a small condition × time interaction. Taking potential dropouts into account, a total sample of 162 patients was assigned to one of the three treatment groups by a stratified randomization process.

MLMs were used to investigate change in symptom severity and therapeutic alliance, respectively. Specifically, we used three‐level models, considering the repeated assessments (Level 1) as nested within patients (Level 2), which are nested in therapists (Level 3). For all research questions, model fit of the competing models was compared by means of likelihood ratio tests for nested models and the Akaike Information Criterion (smaller values indicate better model fit). We used Level 1 pseudo *R*² according to Xu (2003) as a measure of effect size. This estimate represents the amount of Level 1 variance accounted for by the model, that is, the amount of within‐patient fluctuations in symptom severity/therapeutic alliance explained by the model's predictors. Formal model descriptions are reported in the Supporting Information Appendix. We conducted intention‐to‐treat (ITT) and per protocol analyses with and without a priori identified covariates (age, gender, comorbidity, disorder group, and allegiance). As results were conceptually identical, for parsimonious reasons, we present only results of the ITT analyses including the covariates.

We predicted symptom severity ratings obtained at Pre, S5, S15, and S25 from the treatment condition (TAU+M, TAU+PMR, and TAU) and time. The condition variable was represented by two dummy variables comparing the mindfulness condition to the PMR condition (SIIMEvsPMR) and the mindfulness condition to the TAU condition (SIIMEvsTAU), respectively. Time was coded as 1, 2, 3, and 4 and log transformed before the analyses, in line with previous research showing a faster initial change in symptom severity followed by a more decelerated change toward the end of therapy (Nissen‐Lie, Monsen, Ulleberg, & Rønnestad, 2013). We further controlled for gender, age, disorder group, comorbidity, and allegiance. Age and allegiance were centered on the sample mean, and gender, disorder group, and comorbidity were entered as dichotomous variables.

We used a multivariate MLM to simultaneously model a change in alliance ratings from the patient and therapist perspectives (Baldwin et al., 2014; Mander et al., 2017b). Perspective (a dichotomous variable representing whether the alliance rating was based on the therapist or patient perspective), condition (coded with the same two dummy variables as in the previous models), and time (session number) were added as main predictors in these models. The continuous session number (ranging from 1 to 25) was log transformed before the analyses. In addition to the main effects of these predictors, two‐way interactions were added in a second model, followed by the perspective × condition × time interaction in the final model. Gender, age, disorder group, comorbidity, and allegiance were entered as covariates.

Furthermore, we examined whether condition moderates the association between changes of therapeutic alliance across the therapy sessions with changes in symptom severity. To that end, we first extracted person‐specific regression coefficients for change in therapeutic alliance from both the patient and therapist perspective. Specifically, we set up a multivariate MLM with alliance (rated from the patient and therapist perspective) as a dependent variable, and perspective, time (logarithmized session number), and the perspective × time interaction as fixed and random effects predictors (random effects were estimated on the patient and the therapist level, respectively). From this model, we extracted person‐specific regression coefficients for each patient for change in alliance from patient and therapist perspective (see Supporting Information Appendix for details). Hence, each patient’s trajectories are described by four parameters (intercept and change for ratings from patient and therapist perspective, respectively). These patient level predictors were then entered into the MLM, predicting symptom severity. We examined the moderating effect of condition for the association between these change parameters and change in symptom severity by testing the condition × change parameter × time (Pre through S25) interactions.

Finally, on the basis of the guidelines of Jacobson and Truax (1991), we compared response and remission rates across the three study groups. Patients with a reliable change index ≥ 1.96 were considered responders. Patients who additionally had a nonpathological posttreatment score in the relevant outcome measure were considered remitters. All statistical analyses were conducted using R version 3.4.0 (R Core Team, 2017). The MLM procedures were conducted using the R package nlme version 3.1‐13 (Pinheiro, Bates, DebRoy, Sarkar, & R Developmental Core Team, 2018). For all statistical tests, we used a conventional *α*‐level of 0.05 (two‐sided).

## 3 RESULTS

### 3.1 Descriptive analyses

Altogether, 3130 sessions were assessed. Except for employment status, conditions did not differ regarding baseline characteristics (see Table 1). Participants had mostly no prior experience in mindfulness or PMR. A total of 47.9% had at least one comorbid disorder. The main comorbidity of patients with anxiety as a primary diagnosis was a depressive disorder (46.9%). The main comorbidities of patients with depression as primary diagnoses were personality disorders (7.7%), substance use disorders (7.7%), and eating disorders (5.1%). Pre–post effect sizes using Cohen's *d* (Cohen, 1988) for symptoms, alliance ratings, and dispositional mindfulness were moderate to large and comparable across conditions (see Table 2).

**Table 2:** Means, standard deviations (in parentheses) and effect sizes of primary and secondary outcomes

	Entire sample	TAU+M	TAU+PMR	TAU
GSI				
Pretreatment	1.45 (0.67)	1.51 (0.59)	1.37 (0.76)	1.46 (0.67)
Session 5	1.11 (0.64)	1.17 (0.62)	1.04 (0.59)	1.12 (0.69)
Session 15	1.03 (0.66)	1.05 (0.64)	0.98 (0.61)	1.06 (0.73)
Session 25	0.91 (0.68)	0.97 (0.69)	0.77 (0.61)	0.95 (0.73)
ES [95% CI]	0.80 [0.55; 1.06]	0.85 [0.41; 1.29]	0.85 [0.38; 1.33]	0.73 [0.31; 1.16]
BDI‐score				
Pretreatment	26.08 (11.19)	28.91 (9.49)	24.17 (12.27)	24.96 (11.40)
Session 5	20.48 (11.60)	22.56 (11.10)	19.47 (12.24)	19.26 (11.46)
Session 15	18.03 (11.23)	18.94 (11.13)	17.56 (10.90)	17.55 (11.79)
Session 25	16.19 (12.40)	18.90 (12.42)	14.50 (11.82)	15.01 (12.67)
ES [95% CI]	0.84 [0.59; 1.10]	0.92 [0.48; 1.36]	0.80 [0.33; 1.27]	0.83 [0.40; 1.25]
BAI‐score				
Pretreatment	18.54 (11.21)	19.72 (9.94)	17.69 (12.14)	18.12 (11.67)
Session 5	17.13 (10.62)	19.29 (9.95)	14.62 (10.57)	17.18 (11.04)
Session 15	15.69 (10.69)	17.27 (11.24)	13.97 (9.72)	15.64 (10.94)
Session 25	13.88 (10.37)	15.26 (10.88)	12.03 (9.98)	14.03 (10.19)
ES [95% CI]	0.43 [0.18; 0.68]	0.43 [0.00; 0.86]	0.50 [0.04; 0.97]	0.37 [−0.05; 0.80]
KIMS‐score				
Pretreatment	1.96 (0.39)	1.90 (0.36)	1.93 (0.44)	2.05 (0.38)
Session 5	1.98 (0.44)	1.91 (0.44)	1.98 (0.52)	2.06 (0.37)
Session 15	2.04 (0.46)	2.05 (0.45)	1.97 (0.46)	2.10 (0.48)
Session 25	2.11 (0.49)	2.09 (0.49)	2.09 (0.45)	2.16 (0.54)
ES [95% CI]	0.35 [−0.10; 0.60]	0.44 [0.02; 0.87]	0.36 [−0.10; 0.82]	0.24 [−0.17; 0.67]
WAI‐patient				
Session 1–5	6.04 (0.65)	6.02 (0.52)	5.98 (0.80)	6.11 (0.63)
Session 6–15	6.26 (0.67)	6.25 (0.55)	6.24 (0.75)	6.27 (0.71)
Session 16–25	6.33 (0.71)	6.31 (0.58)	6.25 (0.95)	6.42 (0.60)
ES [95% CI]	0.43 [0.19; 0.68]	0.52 [0.10; 0.95]	0.31 [−0.13; 0.76]	0.51 [0.10; 0.92]
WAI‐therapist				
Session 1–5	5.75 (0.52)	5.82 (0.44)	5.70 (0.59)	5.72 (0.53)
Session 6–15	5.97 (0.52)	6.02 (0.48)	5.95 (0.48)	5.94 (0.59)
Session 16–25	6.00 (0.60)	6.11 (0.47)	5.90 (0.71)	5.98 (0.61)
ES [95% CI]	0.46 [0.21; 0.70]	0.63 [0.20; 1.06]	0.32 [−0.13; 0.77]	0.46 [0.06; 0.88]

*Note*. BAI: Beck Anxiety Inventory; BDI: Beck Depression Inventory; CI = Confidence Interval; ES: effect size for the pre–post difference (Cohen’s *d*); GSI: Global Severity Index of Brief Symptom Inventory; KIMS: Kentucky Inventory of Mindfulness Skills; PMR: progressive muscle relaxation; TAU: treatment‐as‐usual; WAI: Working Alliance Inventory, mean scores for the three therapy segments; entire sample: 118 ≤ *N* ≤ 142; TAU+M sample: 41 ≤ *n* ≤ 49; TAU+PMR sample: 34 ≤ *n* ≤ 44; TAU sample: 43 ≤ *n* ≤ 49.

### 3.2 Treatment integrity

Therapy sessions of all study participants were included in the adherence and competence analyses. A total of 274 videotapes were assessed by three Master’s level clinical psychologists. Raters received a 15‐hr training course on how to apply adherence and competence measures and were regularly supervised. The videotape of the first session of all participants was assessed. In addition, videotapes of Sessions 5, 15, and 25 of a sample of 20% from each treatment condition were assessed. Further, all participants completed measures on subjective adherence ratings in Sessions 1, 5, 15, and 25.

#### 3.2.1 Adherence to the study interventions

Mindfulness adherence ratings by observers (*M* = 3.86; *SD* = 0.41), as well as subjective adherence ratings by patients (*M* = 2.90; *SD* = 0.59) and therapists (*M* = 2.86; *SD* = 0.52), were high. In addition, the perceived quality of mindfulness implementation reflected in PQ‐M mean scores (patients: *M* = 2.66; *SD* = 0.50; therapists: *M* = 2.88; *SD* = 0.44) and the in‐session therapeutic presence reflected in TPI mean scores (patients: *M* = 3.01; *SD* = 0.54; therapists: *M* = 3.25; *SD* = 0.32) were high, further indicating good subjective adherence to mindfulness. PMR adherence ratings by observers (*M* = 3.98; *SD* = 0.10), as well as subjective adherence ratings by patients (*M* = 2.99; *SD* = 0.60) and therapists (*M* = 2.93; *SD* = 0.59) were high. None of the participants performed exercises or components of exercises from the not assigned condition. None of the participants of the TAU performed SIIME or PMR at the beginning of therapy sessions. This indicates that the interventions were applied according to the protocol.

#### 3.2.2 Applied CBT techniques

We found no significant differences across the three study conditions in the subjective ratings of the therapists related to the intensity and frequency of the application of relevant CBT techniques (psychoeducation, behavioral activation, cognitive restructuring, and exposure therapy, functional analysis, and relationship techniques), all *F*s (2,139) ≤ 2.12; all *p*s ≥ 0.124.

#### 3.2.3 Therapists’ competence

The CTS mean score did not differ significantly between the TAU+M (M = 4.80; *SD* = 0.66), the TAU+PMR (*M* = 4.71; *SD* = 0.95), and the TAU (*M* = 4.82; *SD* = 0.25) condition, *F* (2,27) = 0.08; *p* = 0.926. These results also indicate that the level of therapist competence was satisfactory in all three treatment conditions according to Shaw et al. (1999) and Weck, Neng, Richtberg, Jakob, and Stangier (2015).

#### 3.2.4 Patients’ and therapists’ allegiance

From the patient perspective, there were no significant differences in allegiance to the session‐introducing interventions in TAU+M (*M* = 2.45; *SD* = 1.00), TAU+PMR (*M* = 2.25; *SD* = 1.00), and TAU (*M* = 2.35; *SD* = 0.99), *F*(2, 138) = 0.47, *p* = 0.625. However, from the therapist perspective, allegiance to the session‐introducing intervention differed between the three conditions, *F*(2, 139) = 23.50, *p* < 0.001. A post hoc Tukey test showed that therapist allegiance was significantly lower in the TAU+PMR condition (*M* = 1.48; *SD* = 0.53) than in the TAU+M (*M* = 2.25; *SD* = 0.73) and TAU condition (*M* = 2.30; *SD* = 0.64), all *p*s < 0.001. Regarding allegiance to the offered treatment, conditions differed neither from the patient perspective (TAU+M: *M* = 6.59; *SD* = 1.54*;* TAU+PMR: *M* = 6.92; *SD* = 1.43*;* TAU: *M* = 6.83; *SD* = 1.48*; F*(2, 137) = 0.61, *p* = 0.545), nor from the therapist perspective (TAU+M: *M* = 6.36; *SD* = 1.25*;* TAU+PMR: *M = *6.33; *SD* = 1.27*;* TAU: *M* = 6.35; *SD* = 1.02*; F*(2, 139) = 0.01, *p* = 0.992).

### 3.3 Is there a stronger symptom reduction in TAU+M versus TAU+PMR and TAU?

In the first set of analyses, we examined whether the three groups differed in the change of GSI scores across therapy. To that end, we set up models of increasing complexity. The first model (Model 0 in Table 2) had only random intercepts on the person level (Level 2) and therapist level (Level 3); no predictors were included. Results showed that only 0.9% of variation in symptom severity could be attributed to differences between therapists, whereas the largest proportion (73.0%) could be attributed to differences between patients. In the second model, the main effects of the covariates (age, gender, comorbidity, disorder group, and allegiance) were added to the main effects of condition and (logarithmized) time. Symptom severity decreased across the four measurement occasions, *b* = −0.150, *p* < 0.001. The model explained 35.5% of the within‐patient variance in symptom severity. In the final model, we examined if the change in symptom severity differed between the three conditions (TAU+M, TAU+PMR, TAU) by adding the condition × time interaction. Model fit did not improve, *χ*²(2) = 0.09, *p* = 0.958, suggesting that the change in symptom severity did not differ between the three conditions. Results for BDI and BAI were conceptually identical with symptoms showing a significant decrease over time (BDI: *b* = −4.466, *p* < 0.001; BAI: *b* = −3.645, *p* < 0.001), which was comparable across conditions as indicated by no improved model fit when adding the condition × time interaction, *χ*²(2) ≤ 1.24, *p*s ≥ 0.539. KIMS scores significantly increased over time, *b* = 0.119, *p* < 0.001, but again, model fit did not improve when adding the condition × time interaction, *χ*²(2) = 2.16, *p* = 0.340. Consequently, the results falsified our assumption that there is a stronger symptom reduction in the mindfulness condition (Hypothesis 1).

### 3.4 Is there a stronger alliance increase in TAU+M versus TAU+PMR and TAU?

**Figure 2:**
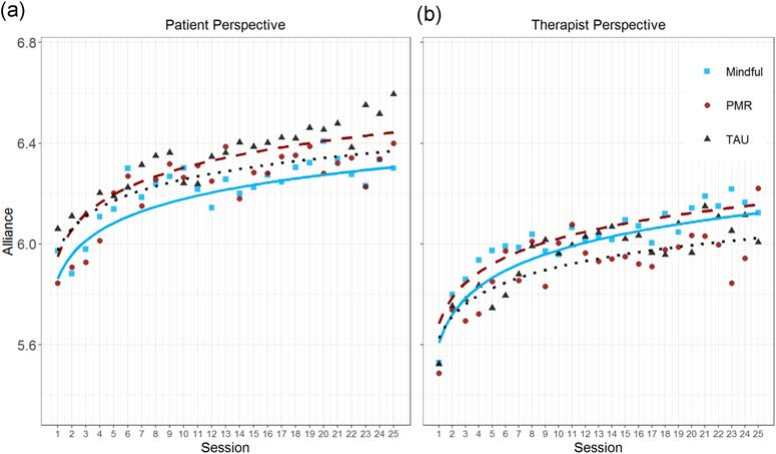
Empirical means (symbols) and estimated trajectories (lines) of the therapeutic alliance, separately for patients in the mindfulness condition (solid blue line, ■), patients in the PMR condition (dashed red line, •), and patients in the TAU condition (dotted black line, ▲). Ratings from the patient perspective are depicted in the left panel, and ratings from the therapist perspective in the right panel. Further information on the trajectories can be found in Table 3 [Color figure can be viewed at wileyonlinelibrary.com]

Fluctuations in therapeutic alliance across the 25 therapeutic sessions were estimated via multivariate MLMs, allowing for simultaneously estimating the trajectories in the ratings from the patient and therapist perspective. Reliabilities of within‐person fluctuations in therapeutic alliance were estimated via McDonald’s ω in a multilevel confirmatory factor analysis (Geldhof, Preacher, & Zyphur, 2014). Results suggested good internal consistency of the 12 items rated from the patient perspective, within‐person ω = 0.86, and the therapist perspective, within‐person ω = 0.86, respectively. In the first model (Model 0 in Table 2), only the dichotomous predictor perspective was entered (as fixed effect and random effect). Across all sessions, therapeutic alliance was rated lower from the therapist perspective than from the patient perspective, *b* = −0.279, *p* < 0.001. Adding the main effects of the covariates, condition, and (logarithmized) time (Model 1) yielded a significant increase across therapy, *b* = 0.143, *p* < 0.001. Adding all two‐way interactions between perspective, condition, and time improved model fit significantly, *χ*²(13) = 165.25, *p* < 0.001 (Model 2). However, none of the five additional fixed effects were significant, *p* > 0.211 for all, suggesting that this increase in model fit might be attributed to the existence of meaningful interindividual differences in the differential change of alliance rated from the two perspectives. Hence, although on average there were no differences between the rates of change in alliance as rated from the therapist and patient perspective, respectively (indicated by the nonsignificant time × perspective interaction), patients and therapists differed in the degree to which there were differences between these two trajectories. We tested whether these differences could be explained by a treatment condition by including the condition × time × perspective interaction (Model 3). This did, however, not improve model fit, *χ*²(2) = 0.853, *p* = 0.653. Consequently, the results falsified our assumption that there is a stronger increase in therapeutic alliance in the mindfulness condition (Hypothesis 2). Figure 2 depicts the trajectories in alliance across therapy sessions separately for the three conditions and for the patient and therapist perspective, respectively.

**Table 3:** Results of MLM growth curve analyses: Fixed and random effect predictors of symptom severity and therapeutic alliance

	Symptom Severity			Alliance			
	Model 0	Model 1	Model 2	Model 0	Model 1	Model 2	Model 3
	Fixed effects			Fixed effects			
Intercept	1.04***	1.03***	1.02***	6.23***	6.00***	5.91***	5.93***
	(0.050)	(0.120)	(0.123)	(0.055)	(0.093)	(0.112)	(0.116)
Perspective^a^	–	–	–	−0.279***	−0.275***	−0.213*	−0.254*
				(0.056)	(0.056)	(0.102)	(0.120)
Age	–	0.004	0.004	–	0.001	0.000	0.000
		(0.004)	(0.004)		(0.002)	(0.003)	(0.003)
Gender^b^	–	−0.155	−0.155	–	−0.147*	−0.140*	−0.139*
		(0.095)	(0.095)		(0.064)	(0.064)	(0.063)
Comorbidity^c^	–	0.339***	0.339***	–	−0.027	−0.023	−0.028
		(0.096)	(0.096)		(0.066)	(0.065)	(0.065)
Disorder^d^	–	0.062	0.062	–	−0.011	−0.006	−0.006
		(0.096)	(0.096)		(0.066)	(0.065)	(0.065)
Allegiance (patient)	–	−0.046	−0.046	–	0.059	0.060	0.060
		(0.048)	(0.048)		(0.032)	(0.032)	(0.032)
Allegiance (therapist)	–	−0.070	−0.070	–	0.083	0.097	0.097
		(0.075)	(0.076)		(0.060)	(0.059)	(0.058)
SIIMEvsPMR	–	−0.102	−0.093	–	0.023	0.118	0.088
		(0.129)	(0.137)		(0.023)	(0.137)	(0.151)
SIIMEvsTAU	–	0.061	0.057	–	−0.014	0.135	0.113
		(0.113)	(0.122)		(0.078)	(0.127)	(0.141)
Time	–	−0.150***	−0.147*	–	0.143***	0.148***	0.138***
		(0.034)	(0.058)		(0.016)	(0.028)	(0.032)
Time × Perspective	–	–	–		–	0.005	0.022
						(0.024)	(0.037)
Time × SIIMEvsPMR	–	–	−0.017	–	–	−0.002	0.015
			(0.085)			(0.035)	(0.046)
Time × SIIMEvsTAU	–	–	0.007	–	–	−0.028	−0.015
			(0.082)			(0.034)	(0.045)
Perspective × SIIMEvsPMR	–	–	–	–	–	−0.076	−0.011
						(0.114)	(0.166)
Perspective × SIIMEvsTAU	–	–	–	–	–	−0.141	−0.095
						(0.112)	(0.163)
Perspective × Time × SIIMEvsPMR	–	–	–	–	–	–	−0.029
							(0.052)
Perspective × Time × SIIMEvsTAU	–	–	–	–	–	–	−0.021
							(0.050)
	Random effects (variances)			Random effects (variances)			
Intercept (Level 3)	0.004	0.008	0.008	0.010	0.018	0.025	0.012
Perspective (Level 3)	–	–		0.049	0.044	0.085	0.063
Time (Level 3)	–	0.000	0.000	‐	0.002	0.001	0.001
Time × Perspective (Level 3)	–	–	–	–	–	0.007	0.006
Intercept (Level 2)	0.304	0.271	0.271	0.395	0.344	0.409	0.423
Perspective (Level 2)	–	–	–	0.270	0.276	0.486	0.499
Time (Level 2)	–	0.086	0.085	–	0.024	0.040	0.040
Time × Perspective (Level 2)	–	–	–	–	–	0.039	0.040
Residual variance (Severity)	0.109	0.070	0.070	–	–	–	–
Residual variance (patient perspective)	–	–	–	0.157	0.127	0.120	0.120
Residual variance (therapist perspective)	–	–	–	0.181	0.152	0.144	0.144
*R*² (Level 1; severity)	–	35.5%	35.5%	–	–	–	–
*R*² (Level 1; patient perspective)	–	–	–	–	19.0%	23.5%	23.5%
*R*² (Level 1; therapist perspective)	–	–	–	–	16.1%	20.6%	20.6%
AIC	681.952	624.342***	628.256	7616.41	6719.10***	6579.85***	6583.00

*Note*. Significance values of AIC indicate results from a likelihood ratio test comparing the current model to the previous model. Continuous predictors (age and allegiance) were centered on the sample mean. Time was coded as 1, 2, 3, and 4 (symptom severity) and 1–25 (alliance), respectively, and log transformed before the analyses.Table depicts point estimates (standard errors in parentheses). SIIMEvsPMR = treatment‐as‐usual+mindfulness condition versus treatment‐as‐usual+progressive muscle relaxation condition; SIIMEvsTAU = treatment‐as‐usual+mindfulness condition versus treatment‐as‐usual. AIC: Akaike Information Criterion; PMR: progressive muscle relaxation; SIIME: session‐introducing intervention with mindfulness element; TAU: treatment‐as‐usual. *N* = 48 therapists, 141 patients, 531 observations (symptom severity), 6260 observations (alliance).

^a^ 0 = patient perspective; 1 = therapist perspective.

^b^ 0 = female; 1 = male.

^c^ 0 = no comorbidity; 1 = comorbid disorder.

^d^ 0 = anxiety; 1 = depression.

* *p* < 0.05.

** *p* < 0.01.

*** *p* < 0.001

### 3.5 Is mindfulness a significant moderator of the alliance–outcome association?

Furthermore, we examined the potential moderating effects of the mindfulness condition on the alliance–outcome association. More specifically, we investigated whether the association of WAI change trajectories and GSI change trajectories was moderated by the treatment condition. We used the person‐level variables representing a change in therapeutic alliance (intercept and change for ratings from the patient and therapist perspective, respectively) as predictors of change in symptoms. Adding the person‐specific change parameters of alliance rated from the patient perspective as main effects and in interaction with time (Pre through S25) improved model fit, *χ*²(4) = 26.12, *p* < 0.001. Inspection of the fixed effects revealed a significant main effect of the intercept: patients with relatively higher intercepts (i.e., higher estimated alliance at the first session) reported less severe symptoms at pre, *b* = −0.248, *p* = 0.004. In addition, there was a significant main effect of a change in alliance and a significant change in the alliance × time interaction: patients who reported stronger increases in alliance across therapy had lower symptom ratings at pre, *b* = −0.733, *p* = 0.020, and reported a stronger symptom decrease across therapy, *b* = −0.480, *p* = 0.018. Adding the interactions with condition did not improve model fit, *χ*²(8) = 4.30, *p* = 0.829, suggesting that condition does not moderate the association between a change in therapeutic alliance (rated from the patient perspective) and a change in symptom severity across therapy. There were also no moderating effects when replacing the change parameters with the respective parameters from the therapist perspective or running the analyses with the BAI and BDI, all *χ*²(8) < 8.92, all *p*s > 0.349. Consequently, the results falsified our assumption that the mindfulness condition is a significant moderator of the alliance–outcome association (Hypothesis 3).

### 3.6 Are there significant moderators of mindfulness effects?

In the final set of analyses, we examined whether patients with specific characteristics benefit more from the mindfulness condition. Therefore, we additionally tested potential moderator variables. Neither mindfulness‐related variables (dispositional mindfulness, prior mindfulness experience, intensity of current mindfulness practice, allegiance) nor demographic and diagnostic variables (sex, age, diagnosis, comorbidity) moderated treatment effects, as indicated by a nonsignificant three‐way interaction with time and condition, all *p*s ≥ 0.122. Hence, we found no significant moderating variables. This partly falsified our fourth hypothesis: The findings contradicted our expectations that mindfulness‐related variables would show significant moderating effects (Hypothesis 4a), but were in line with our assumption that demographic and diagnostic variables would not be significant moderators (Hypothesis 4b).

### 3.7 Response and remission

We assessed response and remission based on observer ratings (HAM‐D/HAM‐A; SCID) and based on self‐ratings (GSI). On the basis of HAM‐D and HAM‐A scores, we identified 56% responders in TAU+M, 53% responders in TAU+PMR, and 53% responders in TAU. We identified 39% remitters in TAU+M, 40% remitters in TAU+PMR, and 41% remitters in TAU. On the basis of SCID ratings, we identified 49% remitters in TAU+M, 52% remitters in TAU+PMR, and 53% remitters in TAU. Finally, we also analyzed response and remission based on the GSI. We identified 53% responders in TAU+M, 57% responders in TAU+PMR, and 51% responders in TAU. We identified 29% remitters in TAU+M, 27% remitters in TAU+PMR, and 31% remitters in TAU. There were no differences in response and remission rates across TAU+M, TAU+PMR, and TAU, all *χ*²(2) < 0.32, all *p*s ≥ 0.852.

## 4 DISCUSSION

In the current study, we investigated whether session‐introducing mindfulness in individual therapy under routine conditions of CBT training has beneficial effects on therapeutic alliance and outcome in a sample of 162 patients with anxiety and depression. We designed an RCT with the three conditions TAU+M versus TAU+PMR versus TAU. Contrary to our expectations, TAU+M did not result in stronger effects on general symptom severity and therapeutic alliance as compared with TAU+PMR and TAU.

Contrary to our expectation, the results of our strictly experimental study design showed no stronger symptom reduction in the mindfulness condition (Hypothesis 1). The treatment effects of all three conditions were of similar strengths and comparable with the effectiveness of routine psychotherapy in meta‐analyses (McAleavey et al., 2017). The results were conceptually identical when we ran the MLM models using the primary (BSI) and the secondary (BDI and BAI) outcome measures. Most studies investigating treatment components administered the added interventions using extra sessions, confounding dose effects with treatment effects (Bell, Marcus, & Goodlad, 2013; Cuijpers et al., 2017). Of note, in our study, we directly integrated SIIME and PMR into the sessions to match overall therapy time and to not confound potential effects with extra treatment time. We applied a strong experimental design with the session‐introducing intervention as the only difference in the three treatment conditions, and strictly assessed adherence, competence, and allegiance. We identified high subjective and external rater adherence to the session‐introducing interventions with no significant differences between the treatment conditions. Furthermore, we found no differences in competence, treatment allegiance, and intensity of applied CBT techniques in the three study conditions. Only allegiance to session introduction from the therapist perspective was lower for TAU+PMR versus TAU+M and TAU. The lower PMR allegiance of therapists might be explained by less positive evaluations of PMR versus SIIME and TAU in posttreatment interviews: Therapists perceived PMR as rather “not modern,” “uninteresting,” and “boring” in comparison to the other two treatment conditions. This might have resulted in lower allegiance ratings. We also found no significant differences in the three treatment conditions related to response and remission rates based on HAM‐D, HAM‐A, and SCID observer ratings. Thus, our results indicate that TAU+M, TAU+PMR, and TAU might be similarly beneficial for treatment outcome in anxiety and depression.

Contrary to our expectation, TAU+M did not show stronger effects related to the therapeutic alliance (Hypothesis 2). In all three treatment conditions, patients and therapists showed positive alliance ratings that increased across the course of therapy with medium effect sizes. There were no significant differences in the strengths of alliance ratings or in the increase of alliance ratings across the course of therapy. Furthermore, we explored whether the alliance–outcome association was different in the three treatment conditions. Therefore, we conducted an MLM moderator analysis. We did not identify differences in the alliance–outcome association across the three treatment conditions. Consequently, the results falsified our prediction that mindfulness is a significant moderator of the alliance–outcome association (Hypothesis 3). As in most studies (Flückiger, Del Re, Wampold, & Horvath, 2018; Horvath et al., 2011), alliance ratings were high, resulting in little potential opportunities for differential alliance effects in the three treatment conditions. This could in part explain the absent mindfulness effect in our study. Furthermore, our results are in line with research on moderating variables of the alliance–outcome association. Meta‐analyses identified a significant overall alliance–outcome relation of *r* = 0.27 that is ubiquitous, irrespective of treatment type, rater perspective, time of alliance assessment, or outcome measure (Flückiger, Del re, Wampold, Symonds, & Horvath, 2012; Horvath et al., 2011). Of note, none of the moderators in these meta‐analyses were experimentally manipulated variables; therefore, causal statements are not warranted (Flückiger et al., 2012; Mander et al., 2017b). Thus, our results add to the robust effect of the therapeutic alliance irrespective of moderating variables, even experimentally manipulated variables that theoretically should result in beneficial alliance effects. However, other research showed that for example alexithymia, symptom feedback, and an integrative therapy approach might have differential effects on the alliance–outcome association (Probst et al., 2017; Zilcha‐Mano & Errázuriz, 2015). Hence, more research is needed to better understand potential moderating variables of the alliance–outcome association.

The results of our study are of importance for routine practice, as many practitioners directly integrate mindfulness into their individual therapies without an empirical basis for whether this component actually improves outcomes (Crane, 2016; Dimidjian & Segal, 2015). Specifically, our study results deliver preliminary empirical evidence that mindfulness has no statistically significant additional effects in individual CBT for anxiety and depression under routine conditions, but also has no adverse effects (Michalak et al., 2015). Possibly, specific subsamples of patients benefit from added mindfulness, whereas other benefit more from standard treatment (Shallcross et al., 2015). In the current study, neither sex, age, diagnosis, comorbidity, dispositional mindfulness, prior mindfulness experience, intensity of current mindfulness practice, nor allegiance moderated treatment effects. This partly falsified our fourth hypothesis: The findings contradicted our expectations that mindfulness‐related variables would show significant moderating effects (Hypothesis 4a), but were in line with our assumption that demographic and diagnostic variables would not be significant moderators (Hypothesis 4b). However, the power of our current study might have been too small to identify potential subgroup effects. As psychotherapeutic treatments further develop into individualized intervention programs (Lutz, Zimmermann, Müller, Deisenhofer, & Rubel, 2017), future studies with larger power should investigate this aspect in more detail. Finally, the absence of outcome effects between the conditions does not necessarily imply that there were no meaningful differences in the way CBT was applied. This reasoning was supported by qualitative interviews conducted with all patients and therapists at the end of therapy (for further details, see Mander et al., 2017a). The interviews indicated that TAU+M had positive effects on the sessions as the SIIME helped to come into a state of openness and present moment awareness. The physical relaxation induced by PMR helped patients to open up with their difficulties and therapists to concentrate better when trying to understand the problems of their patients. TAU had a positive impact on the sessions as patients and therapists were free to arrange the start of therapy the way that seemed most consistent for them. Thus, although the conditions were equally effective, the reasons for achieving this outcome may differ.

A large part of the current therapist sample started mindfulness training with the workshop presented at the beginning of the study. Hence, according to the framework for an MBI teacher training program, the participants were in the first stage of foundational training (Crane, Kuyken, Hastings, Rothwell, & Williams, 2010). Experts postulate that therapists working with mindfulness should have at least 2 years of experience of daily mindfulness practice (Michalak et al., 2012; Segal et al., 2013). Possibly, MBIs can only be applied effectively by these experienced therapists (Crane et al., 2010; Kuyken et al., 2016). In fact, in our study, increases of patient KIMS scores were not stronger in TAU+M versus TAU+PMR and TAU. Possibly, this lack of mindfulness increase is attributable to the delivery of mindfulness exercises by inexperienced therapists. However, first studies found that therapists’ mindfulness competence and experience are not significantly associated with patient outcomes (Huijbers et al., 2017). Nevertheless, these results are only preliminary. Future studies should compare therapists with high MBI experience with therapists with little mindfulness experience in adequately powered samples (Dimidjian & Segal, 2015).

The current study has several limitations. First, as we investigated routine therapy, treatment was not manualized and several patients suffered from comorbid disorders. This might have resulted in internal validity issues. To address this aspect, we controlled for potential confounding variables in the MLM analyses. In addition, routine care investigations have high external validity and are, thus, relevant for clinical practitioners. Second, we applied only self‐report outcome measures for the repeated assessments. We only assessed observer ratings using SCID, HAM‐A, and HAM‐D at the beginning and end of treatment and did not have the opportunity to apply these ratings at all measuring times. Future studies should apply multiple observer ratings to investigate whether our effects generalize to other outcome rating perspectives. Third, 15% of TAU+M, 22% of TAU+PMR, and 13% of TAU were dropouts, indicating that treatment was not accepted by all patients. According to a meta‐analysis, 19.7% of study participants drop out in RCTs (Swift & Greenberg, 2012). Hence, dropout rate in the current study was comparable and the acceptability of treatment can be considered as satisfactory (Swift & Greenberg, 2012). Finally, mindfulness as a component directly integrated into individual therapy sessions might be too weak to result in additional outcome effects (Bell et al., 2013; Cuijpers et al., 2017). However, the procedure of the current study reflects the way that practitioners integrate mindfulness into individual therapy routines (Dimidjian & Segal, 2015). Hence, empirical findings are of importance to understand whether this clinical practice has positive or negative effects on treatment (Blanck et al., 2017).

Our study delivers first evidence that mindfulness as an add‐on component in individual therapy has similar effects as PMR and TAU on therapeutic alliance and clinical symptoms in anxiety and depression. Add‐on mindfulness might not improve individual therapy related to alliance and outcome.

## Supporting information

Supporting Information

## APPENDIX 1

### Experimental condition: Session‐introducing intervention with mindfulness elements

You are sitting in the chair in a position that feels comfortable to you. You sit upright; your feet are flat on the floor. Your hands are resting on your upper legs. If it makes you feel good, you can allow your eyes to close gently [5‐second break].

Throughout the exercise, please take an open‐minded non‐judgmental stance vis‐à‐vis everything you experience in the here and now [5‐second break]. Focus all of your attention on your breath. Observe as the air is inhaled and exhaled [15‐second break].

Now come into awareness with the physical sensations of your breath. Try to simply accept all of the experiences as they occur to you without judging them [15‐second break]. By paying attention to your breath you will gradually advance into the here and now. You may now notice how your abdomen is lifted up as you inhale and how it is lowered again as you exhale. [15‐second break].

Keep focusing your attention on your breath and always return to it even when your thoughts begin to wander. Try to encounter everything you experience with a friendly, accepting attitude [40‐second break].

Now, expand your sphere of attention and simply become aware of the feelings in the rest of your body without making any attempt to alter them [15‐second break]. Maybe you will become aware of your physical stance or the expression of your face [15‐second break]. As you are doing this, open up to all of the experiences that unfold in the present moment. [40‐second break].

Next, whenever you are ready, let go of these feelings. Slowly open your eyes with the intention of bringing this attention into the present moment and the rest of the day.

### Control condition: Session‐introducing progressive muscle relaxation exercise

You are sitting in the chair in a position that feels comfortable to you. You sit upright; your feet are flat on the floor. Your hands are resting on your upper legs. If it makes you feel good, you can allow your eyes to close gently [5‐second break].

First, focus your attention on the muscles in both of your arms. Tighten both of your arms simultaneously – now – [5‐second break] – and when you exhale the next time, let go and relax your arms. Focus intensively on the gradual release of tension and the sense of relaxation that ensues [20‐second break].

Now, please focus your attention on the muscles in your face, neck and nape of the neck. Tighten up your face, neck and nape of the neck – all at the same time – now ‐ [5‐second break]. Then, as you exhale again, release the tension and relax. Every time you exhale, allow yourself to relax more deeply and even deeper the next time [20‐second break].

Next, turn your attention on the muscles in your torso. Tighten up your shoulders, back and belly at the same time – now – [5‐second break]. As you exhale again, allow the tension to be released and relax. Allow this release to flow down from the shoulder to the spine and down your entire back every time you inhale or exhale [20‐second break].

At this point, focus your attention on your legs, please. Tighten up both legs simultaneously ‐ now – [5‐second break]. When you exhale again, let go of the tension and relax. Notice the pleasant feeling of relaxation, which gradually spreads through both legs [20‐second break].

In the final exercise, please tension up all muscles in your entire body – now – [5‐second break]. When you exhale again, let all of the tension go and relax. As you breath in and out several times, surrender yourself completely to the sense of relaxation and allow it flow through your whole body [20‐second break].

Next, whenever you are ready, let go of these feelings. Slowly open your eyes with the intention of bringing this relaxation into the present moment and the rest of the day.
