# Supplementary material for: Mindfulness and progressive muscle relaxation as standardized session‐introduction in individual therapy: A randomized controlled trial
Source: J Clin Psychol. 2018 Oct 8;75(1):21–45. doi: 10.1002/jclp.22695 (PMC6826255; doi:10.1002/jclp.22695)
Supplement: Supplementary file 1 — Supporting Information [file JCLP-75-21-s001.docx]

**Rationale for the three study conditions**

**Treatment as usual + mindfulness (TAU + M) - Rationale** Standardised introduction for the first session (Literally read by the therapist, instructions for the therapist are written in *italics*): "During the briefing, you agreed to participate in our study and be randomly assigned to one of three conditions: "mindfulness", "progressive muscle relaxation (PMR)" or "treatment as usual". The ballot has decided that our treatment will follow the condition "mindfulness". Mindfulness is a specific form of attention control that is non-judgmental, intentional, and focused on the current moment. You will probably already have noticed during the diagnostic sessions that both you and I come to the session more or less abruptly from our everyday lives with their usual, often diverse content. So at the beginning of each session, we will do a 5-minute exercise with mindfulness elements to make the transition from everyday life to therapy. This is done via a small speaker (*show speaker*) and is identical for all sessions. The exercise will thus be a kind of session-initiating ritual. The approximately 5-minute mindfulness exercise will give you and me the opportunity to "arrive" properly in the therapy session by directing attention to the current moment.”

**Treatment as usual + PMR (TAU + PMR) - Rationale** Standardised introduction for the first session (Literally read by the therapist, instructions for the therapist are written in *italics*): " During the briefing, you agreed to participate in our study and be randomly assigned to one of three conditions: "mindfulness", "progressive muscle relaxation (PMR)" or "treatment as usual". The ballot has decided that our treatment will follow the condition "PMR". PMR is a procedure that aims to achieve a state of relaxation through the deliberate and conscious growth and relaxation of specific muscle groups. You will probably already have noticed during the diagnostic sessions that both you and I come to the session more or less abruptly from our everyday lives with their usual, often diverse content. So at the beginning of each session, we will do a 5-minute PMR exercise to make the transition from everyday life to therapy. This is done via a small speaker (*show speaker*) and is identical for all sessions. The exercise will thus be a kind of session-initiating ritual. The approximately 5 minutes PMR exercise will give you and me the opportunity to "arrive" properly in the therapy session by activating relaxation.”

**Treatment as usual (TAU) - Rationale** Standardised introduction for the first session (Literally read by the therapist, instructions for the therapist are written in *italics*): "During the briefing, you agreed to participate in our study and be randomly assigned to one of three conditions: "mindfulness", "progressive muscle relaxation (PMR)" or "treatment as usual". The ballot has decided that our treatment will follow the condition "treatment as usual". "Treatment as usual" is the proven treatment at the training centre, which is usually performed here. So there will be no standardised exercise at the beginning of the sessions. Accordingly, we are free to arrange the start of therapy as well as the entire course of therapy in the way that seems the most consistent for us. Thus, the entire 50 minutes of every single therapy session is available for the therapeutic work on your individual issues.”

**Appendix for Multilevel Modeling**

The following equations describe the final models implemented to assess our research questions. The indices *t*, *i*, and *j* in the following sections refer to the measurement occasions (*t*, Level 1), patients (*i*, Level 2), and therapists (*j*, Level 2), respectively.

**Change in Symptom Severity**

The dependent variable symptom severity (SS*_tij_*) was obtained at four measurement occasions (T1 through T4). This variable was predicted by condition, a factor with three levels that was coded as two dummy variables comparing the mindfulness condition to the PMR condition (SIIMEvsPMR*_ti_*) and the mindfulness condition to the TAU condition (SIIMEvsTAU*_ti_*), respectively, and logarithmized time (log_t_tij_). Further, the interaction effects of log time with the two dummies were added, as were the effects of the Level 2 covariates age (age*_ij_*), gender (gender*_ij_*), disorder group (dis*_ij_*), comorbidity (com*_ij_*), patient allegiance (pall*_ij_*) and therapist allegiance (tall*_ij_*). Continuous predictors (age*_ij_*, pall*_ij_*, and tall*_ij_*) were centered on their respective sample means. The intercept and the effect of log_t_tij_ were estimated as random effect on Level 2 and Level 3, and the random effects were allowed to covary within each level (unrestricted G matrices). The resulting multilevel equations are:

| Level 1:  ${SS}_{tij}=$ | $\beta_{0ij}+\beta_{1ij}{\cdot log\_t}_{tij}+\varepsilon_{tij}$ | (A1) |
| --- | --- | --- |
| Level 2 (Patients):  $\beta_{0ij}=$ | $\gamma_{0j}+\gamma_{01}{\cdot SIIME\mathrm{vsPMR}}_{ij}+\gamma_{02}{\cdot SIIME\mathrm{vsTAU}}_{ij}+$  $\gamma_{03}{\cdot age}_{ij}+\gamma_{04}{\cdot gender}_{ij}+\gamma_{05}{\cdot dis}_{ij}+\gamma_{06}{\cdot com}_{ij}+$  $\gamma_{07}{\cdot pall}_{ij}+\gamma_{08}{\cdot tall}_{ij}+\upsilon_{0ij}$ | (A2) |
| $\beta_{1ij}=$ | $\gamma_{1j}+\gamma_{11}{\cdot SIIME\mathrm{vsPMR}}_{ij}+\gamma_{12}{\cdot SIIME\mathrm{vsTAU}}_{ij}+\upsilon_{1ij}$ | (A3) |
| Level 3 (Therapists):  $\gamma_{0j}=$ | $\pi_{00}+\xi_{0j}$ | (A4) |
| $\gamma_{1j}=$ | $\pi_{10}+\xi_{1j}$ | (A5) |

| $\varepsilon_{tij} \sim N(0, \sigma_{\varepsilon}^{2}$) | (A6) |
| --- | --- |
| $\left( \begin{matrix} \upsilon_{0ij} \\ \upsilon_{1ij} \end{matrix} \right)\sim N\left( \left( \begin{matrix} 0 \\ 0 \end{matrix} \right), \left( \begin{matrix} \sigma_{\upsilon_{0}}^{2} & \sigma_{\upsilon_{0},\upsilon_{1}} \\ \sigma_{\upsilon_{0},\upsilon_{1}} & \sigma_{\upsilon_{1}}^{2} \end{matrix} \right) \right)$ | (A7) |
| $\left( \begin{matrix} \xi_{0j} \\ \xi_{1j} \end{matrix} \right)\sim N\left( \left( \begin{matrix} 0 \\ 0 \end{matrix} \right), \left( \begin{matrix} \sigma_{\xi_{0}}^{2} & \sigma_{\xi_{0},\xi_{1}} \\ \sigma_{\xi_{0},\xi_{1}} & \sigma_{\xi_{1}}^{2} \end{matrix} \right) \right)$ | (A8) |

**Change in Therapeutic Alliance**

A multivariate multilevel model was built by ‘stacking’ the responses for alliance rated from the two perspectives into one variable. That is, each patient had up to 50 responses on the dependent variable (25 sessions x 2 perspectives). In addition to the predictors that had been included for symptom severity, therapeutic alliance (TA*_tij_*) was also predicted by the dichotomous variable perspective (persp*_tij_*) coding whether the response corresponds to the rating from the patient perspective (persp*_tij_* = 0) or to the rating from the therapist perspective (persp*_tij_* = 1). Time was coded as continuous session (from 1 to 25) and log transformed prior to the analyses (log_sess_tij_). In addition to the main effects, two-way interactions between persp*_tij_*, log_sess_tij_, and the dummy variables were added, as well as the corresponding three-way interactions. Random effects were estimated for the intercept, persp*_tij_*, log_sess_tij_, and the persp*_tij_* by log_sess_tij_ interaction on the patient level and the therapist level. Again, random effects were allowed to covary within the levels. Separate Level 1 residual variances for the patient and therapist perspective were estimated.

| Level 1:  ${TA}_{tij}=$ | $\beta_{0ij}+\beta_{1ij}{\cdot persp}_{tij}+\beta_{2ij}\cdot{log\_sess}_{tij}+$  $\beta_{3ij}{\cdot(persp}_{tij} \times{log\_sess}_{tij}) +\varepsilon_{tij}$ | (A9) |
| --- | --- | --- |
| Level 2 (Patients):  $\beta_{0ij}=$ | $\gamma_{0j}+\gamma_{01}\cdot{\mathrm{SIIME}\mathrm{vsPMR}}_{ij}+\gamma_{02}\cdot{\mathrm{SIIME}\mathrm{vsTAU}}_{ij}+$  $\gamma_{03}{\cdot age}_{ij}+\gamma_{04}\cdot\mathrm{gender}_{ij}+\gamma_{05}{\cdot dis}_{ij}+\gamma_{06}{\cdot com}_{ij}+$  $\gamma_{07}{\cdot pall}_{ij}+\gamma_{08}{\cdot tall}_{ij}+\upsilon_{0ij}$ | (A10) |
| $\beta_{1ij}=$ | $\gamma_{1j}+\gamma_{11}{\cdot SIIME\mathrm{vsPMR}}_{ij}+\gamma_{12}{\cdot SIIME\mathrm{vsTAU}}_{ij}+\upsilon_{1ij}$ | (A11) |
| $\beta_{2ij}=$ | $\gamma_{2j}+\gamma_{21}\cdot{\mathrm{SIIME}\mathrm{vsPMR}}_{ij}+\gamma_{22}{\cdot SIIME\mathrm{vsTAU}}_{ij}+\upsilon_{2ij}$ | (A12) |
| $\beta_{3ij}=$ | $\gamma_{3j}+\gamma_{31}\cdot{\mathrm{SIIME}\mathrm{vsPMR}}_{ij}+\gamma_{32}{\cdot SIIME\mathrm{vsTAU}}_{ij}+\upsilon_{3ij}$ | (A12) |
| Level 3 (Therapists):  $\gamma_{0j}=$ | $\pi_{00}+\xi_{0j}$ | (A13) |
| $\gamma_{1j}=$ | $\pi_{10}+\xi_{1j}$ | (A14) |
| $\gamma_{2j}=$ | $\pi_{20}+\xi_{2j}$ | (A15) |
| $\gamma_{3j}=$ | $\pi_{30}+\xi_{3j}$ | (A16) |

| $\varepsilon_{tij} \sim\left\{ \begin{matrix} N\left( 0, \sigma_{\varepsilon_{P}}^{2} \right) \mathrm{If}\mathrm{persp}_{tij}= 0 \\ N(0, \sigma_{\varepsilon_{t}}^{2}) \mathrm{If}\mathrm{persp}_{tij}= 1 \end{matrix} \right.$ | (A17) |
| --- | --- |
| $\left( \begin{matrix} \upsilon_{0ij} \\ \begin{matrix} \upsilon_{1ij} \\ \begin{matrix} \upsilon_{2ij} \\ \upsilon_{3ij} \end{matrix} \end{matrix} \end{matrix} \right)\sim N\left( \left( \begin{matrix} 0 \\ \begin{matrix} 0 \\ \begin{matrix} 0 \\ 0 \end{matrix} \end{matrix} \end{matrix} \right), \left( \begin{matrix} \sigma_{\upsilon_{0}}^{2} & \begin{matrix} \sigma_{\upsilon_{0},\upsilon_{1}} & \begin{matrix} \sigma_{\upsilon_{0},\upsilon_{2}} & \sigma_{\upsilon_{0},\upsilon_{3}} \end{matrix} \end{matrix} \\ \begin{matrix} \sigma_{\upsilon_{0},\upsilon_{1}} \\ \begin{matrix} \sigma_{\upsilon_{0},\upsilon_{2}} \\ \sigma_{\upsilon_{0},\upsilon_{3}} \end{matrix} \end{matrix} & \begin{matrix} \begin{matrix} \sigma_{\upsilon_{1}}^{2} & \begin{matrix} \sigma_{\upsilon_{1},\upsilon_{2}} & \sigma_{\upsilon_{1},\upsilon_{3}} \end{matrix} \end{matrix} \\ \begin{matrix} \begin{matrix} \sigma_{\upsilon_{1},\upsilon_{2}} \\ \sigma_{\upsilon_{1},\upsilon_{3}} \end{matrix} & \begin{matrix} \begin{matrix} \sigma_{\upsilon_{2}}^{2} \\ \sigma_{\upsilon_{2},\upsilon_{3}} \end{matrix} & \begin{matrix} \sigma_{\upsilon_{2},\upsilon_{3}} \\ \sigma_{\upsilon_{3}}^{2} \end{matrix} \end{matrix} \end{matrix} \end{matrix} \end{matrix} \right) \right)$ | (A18) |
| $\left( \begin{matrix} \xi_{0j} \\ \begin{matrix} \xi_{1j} \\ \begin{matrix} \xi_{2j} \\ \xi_{3j} \end{matrix} \end{matrix} \end{matrix} \right)\sim N\left( \left( \begin{matrix} 0 \\ \begin{matrix} 0 \\ \begin{matrix} 0 \\ 0 \end{matrix} \end{matrix} \end{matrix} \right), \left( \begin{matrix} \sigma_{\xi_{0}}^{2} & \begin{matrix} \sigma_{\xi_{0},\xi_{1}} & \begin{matrix} \sigma_{\xi_{0},\xi_{2}} & \sigma_{\xi_{0},\xi_{3}} \end{matrix} \end{matrix} \\ \begin{matrix} \sigma_{\xi_{0},\xi_{1}} \\ \begin{matrix} \sigma_{\xi_{0},\xi_{2}} \\ \sigma_{\xi_{0},\xi_{3}} \end{matrix} \end{matrix} & \begin{matrix} \begin{matrix} \sigma_{\xi_{1}}^{2} & \begin{matrix} \sigma_{\xi_{1},\xi_{2}} & \sigma_{\xi_{1},\xi_{3}} \end{matrix} \end{matrix} \\ \begin{matrix} \begin{matrix} \sigma_{\xi_{1},\xi_{2}} \\ \sigma_{\xi_{1},\xi_{3}} \end{matrix} & \begin{matrix} \begin{matrix} \sigma_{\xi_{2}}^{2} \\ \sigma_{\xi_{2},\xi_{3}} \end{matrix} & \begin{matrix} \sigma_{\xi_{2},\xi_{3}} \\ \sigma_{\xi_{3}}^{2} \end{matrix} \end{matrix} \end{matrix} \end{matrix} \end{matrix} \right) \right)$ | (A19) |

**Analysis of Moderating Effects of Condition on the Alliance-Outcome Association**

For this final set of analyses, we first extracted the person specific regression coefficients describing each patient’s alliance trajectories. To that end, we set up a multivariate multilevel model similar to the model described in the previous section, in which only the effects of persp*_tij_*, log_sess*_tij_*, and the persp*_tij_* by log_sess*_tij_* interaction were included in the model (hence, the model is equivalent to the model in the previous section, if all fixed $\gamma$ parameters are set to zero). From the person specific regression coefficients ($\beta_{0ij}$, $\beta_{1ij}$, $\beta_{2ij}$, and $\beta_{3ij})$ we computed four parameters that described the estimated change trajectories in therapeutic alliance from the patient and therapist perspective, respectively: (1) The intercept in therapeutic alliance from patient perspective (intP*_ij_*; corresponding to the estimated therapeutic alliance from the patient perspective of patient *i* at the first session); (2) the intercept in therapeutic alliance from therapist perspective (intT*_ij_*; corresponding to the estimated therapeutic alliance from the therapist perspective of patient *i* at the first session); (3) the slope of therapeutic alliance from the patient perspective (slopeP*_ij_*; corresponding to the estimated rate of change in therapeutic alliance from the patient perspective of patient *i*); and (4) the slope of therapeutic alliance from the therapist perspective (slopeT*_ij_*; corresponding to the estimated rate of change in therapeutic alliance from the therapist perspective of patient *i*). These parameters can be expressed as a function of the person specific regression coefficients:

| $\mathrm{intP}_{ij}$ = $\beta_{0ij}$ | (A20) |
| --- | --- |
| $\mathrm{intT}_{ij}$ = $\beta_{0ij}$+$\beta_{1ij}$ | (A21) |
| $\mathrm{slopeP}_{ij}$ = $\beta_{2ij}$ | (A22) |
| $\mathrm{slopeT}_{ij}$ = $\beta_{2ij}$+$\beta_{3ij}$ | (A23) |

These variables were then entered into the multilevel model predicting symptom severity. Specifically, the main effects of $\mathrm{intP}_{ij}$and $\mathrm{slopeP}_{ij}$ were added together with the two-way interactions with log_t*_tij_* ($\gamma_{13}$ and $\gamma_{14}$) and the dummy coded condition variables SIIMEvsPMR*_ti_* and SIIMEvsTAU*_ti_* ($\gamma_{011}$ to $\gamma_{014}$), as well as the corresponding three-way interactions ($\gamma_{14}$ to $\gamma_{17}$).

| Level 1:  ${SS}_{tij}=$ | $\beta_{0ij}+\beta_{1ij}{\cdot log\_t}_{tij}+\varepsilon_{tij}$ | (A24) |
| --- | --- | --- |
| Level 2 (Patients):  $\beta_{0ij}=$ | $\gamma_{0j}+\gamma_{01}{\cdot SIIME\mathrm{vsPMR}}_{ij}+\gamma_{02}{\cdot SIIME\mathrm{vsTAU}}_{ij}+$  $\gamma_{03}{\cdot age}_{ij}+\gamma_{04}{\cdot gender}_{ij}+\gamma_{05}{\cdot dis}_{ij}+\gamma_{06}{\cdot com}_{ij}+$  $\gamma_{07}{\cdot pall}_{ij}+\gamma_{08}{\cdot tall}_{ij}+$  $\gamma_{09}{\cdot intP}_{ij}+\gamma_{010}{\cdot slopeP}_{ij}+$  $\gamma_{011}{\cdot(intP}_{ij}\times{\mathrm{SIIME}\mathrm{vsPMR}}_{ij})+$  $\gamma_{012}{\cdot(intP}_{ij}\times{\mathrm{SIIME}\mathrm{vsTAU}}_{ij})+$  $\gamma_{013}{\cdot(slopeP}_{ij}\times{\mathrm{SIIME}\mathrm{vsPMR}}_{ij})+$  $\gamma_{014}{\cdot(slopeP}_{ij}\times{\mathrm{SIIME}\mathrm{vsTAU}}_{ij})+\upsilon_{0ij}$ | (A25) |
| $\beta_{1ij}=$ | $\gamma_{1j}+\gamma_{11}{\cdot SIIME\mathrm{vsPMR}}_{ij}+\gamma_{12}{\cdot SIIME\mathrm{vsTAU}}_{ij}+$  $\gamma_{13}{\cdot intP}_{ij}+\gamma_{14}{\cdot slopeP}_{ij}+$  $\gamma_{14}{\cdot(intP}_{ij}\times{\mathrm{SIIME}\mathrm{vsPMR}}_{ij})+$  $\gamma_{15}{\cdot(intP}_{ij}\times{\mathrm{SIIME}\mathrm{vsTAU}}_{ij})+$  $\gamma_{16}{\cdot(slopeP}_{ij}\times{\mathrm{SIIME}\mathrm{vsPMR}}_{ij})+$  $\gamma_{17}{\cdot(slopeP}_{ij}\times{\mathrm{SIIME}\mathrm{vsTAU}}_{ij})+\upsilon_{1ij}$ | (A26) |
| Level 3 (Therapists):  $\gamma_{0j}=$ | $\pi_{00}+\xi_{0j}$ | (A27) |
| $\gamma_{1j}=$ | $\pi_{10}+\xi_{1j}$ | (A28) |
